# Extremophilic Oxidoreductases for the Industry: Five Successful Examples With Promising Projections

**DOI:** 10.3389/fbioe.2021.710035

**Published:** 2021-08-12

**Authors:** Giannina Espina, Joaquín Atalah, Jenny M. Blamey

**Affiliations:** ^1^Fundación Biociencia, Santiago, Chile; ^2^Facultad de Química y Biología, Universidad de Santiago de Chile, Santiago, Chile

**Keywords:** laccase, hydrogenase, glutamate dehydrogenase, superoxide dismutase, catalase, extremozymes

## Abstract

In a global context where the development of more environmentally conscious technologies is an urgent need, the demand for enzymes for industrial processes is on the rise. Compared to conventional chemical catalysts, the implementation of biocatalysis presents important benefits including higher selectivity, increased sustainability, reduction in operating costs and low toxicity, which translate into cleaner production processes, lower environmental impact as well as increasing the safety of the operating staff. Most of the currently available commercial enzymes are of mesophilic origin, displaying optimal activity in narrow ranges of conditions, which limits their actual application under industrial settings. For this reason, enzymes from extremophilic microorganisms stand out for their specific characteristics, showing higher stability, activity and robustness than their mesophilic counterparts. Their unique structural adaptations allow them to resist denaturation at high temperatures and salinity, remain active at low temperatures, function at extremely acidic or alkaline pHs and high pressure, and participate in reactions in organic solvents and unconventional media. Because of the increased interest to replace chemical catalysts, the global enzymes market is continuously growing, with hydrolases being the most prominent type of enzymes, holding approximately two-third share, followed by oxidoreductases. The latter enzymes catalyze electron transfer reactions and are one of the most abundant classes of enzymes within cells. They hold a significant industrial potential, especially those from extremophiles, as their applications are multifold. In this article we aim to review the properties and potential applications of five different types of extremophilic oxidoreductases: laccases, hydrogenases, glutamate dehydrogenases (GDHs), catalases and superoxide dismutases (SODs). This selection is based on the extensive experience of our research group working with these particular enzymes, from the discovery up to the development of commercial products available for the research market.

## Introduction

Enzymes are advantageous biocatalysts that can effectively replace toxic and expensive chemicals, save on energy/resources consumption, and reduce detrimental impact on the environment. Biocatalysis is increasingly gaining interest for application in several industrial processes, as the use of enzymes has great potential for helping establish a bio-based economy, fitting well with the development of highly efficient, sustainable, and eco-friendly industries.

The demand and trade for industrial enzymes is steadily growing. In 2019, the global enzymes market was valued at $8.63 billion, and is expected to reach $14.5 billion by 2027, registering a CAGR (Compound Annual Growth Rate) of 6.5% from 2020 to 2027 (Manjrekar and Wadekar, 2021). Among the different types of enzymes, hydrolases are the largest contributor to the global market, holding approximately two-third share, while oxidoreductase enzymes are the second largest revenue generators (Manjrekar and Wadekar, 2021).

Oxidoreductases are capable of catalyzing redox reactions, where the oxidation, removal of electrons from an electron donor molecule (i.e., reducing agent), occurs with the concurrent reduction of an electron acceptor molecule (i.e., oxidizing agent) given that electrons are not stable in free state. The redox potential is the affinity of a substance to accept electrons and become reduced, and electrons get transferred from substances with lower redox potential to substances with higher redox potential. This transfer of electrons is an energy yielding process and the amount of energy liberated depends on the redox potential difference between the electron donor and acceptor. As this energy is required to maintain the structure and function of living cells, oxidoreductases are very important and are one of the most abundant classes of enzymes within cells, comprising about one third of all the enzymatic activities registered in BRaunschweig Enzyme Database (BRENDA) (Vidal et al., 2018). They are classified as EC 1, according to the Enzyme Commission Number classification system, and due to their vast diversity, they have been further divided into 23 subclasses according to the electron donors and acceptors they use. Table 1 shows some of the different types of oxidoreductases.

**TABLE 1:** Different types of oxidoreductases.

**Type**	**Characteristics**
Oxidases	Catalyze direct transfer of hydrogen from substrates to oxygen. producing water (or in some cases hydrogen peroxide)
	Named as “Donor” oxidase with O_2_ as the acceptor
	Examples: Bilirubin oxidase (EC 1.3.3.5), Monoamine oxidases (MAO) (EC 1.4.3.4), Laccases (EC 1.10.3.2)
	General reaction:
	Substrate_(reduced)_ + O_2_ = Product_(*oxidized*)_ + H_2_O
Dehydrogenases	Catalyze the oxidation of substrates by transferring one or more hydride ions to an acceptor substrate. Named as “donor” dehydrogenase
	Examples: Alcohol dehydrogenase (EC 1.1.1.1), Glutamate dehydrogenases (EC 1.4.1), Hydrogenases (EC 1.12)
	General reaction:
	Substrate1_(__*oxidized*__)_ + Substrate2_(reduced)_ = Product1_(reduced)_ + Product2_(__*oxidized*__)_ + H^+^
Reductases	Catalyze the reduction of a substrate. Named as “acceptor” reductase
	Examples: Glutathione-disulfide reductase (EC 1.8.1.7)
	General reaction:
	Substate + Electron acceptor = Product + Reduced acceptor
Dismutases	Catalyzes a dismutation reaction (or disproportionation) where one compound of intermediate oxidation state converts to two compounds, one of higher and one of lower oxidation states
	Example: Catalase (EC 1.11.1.6), Superoxide dismutase (EC 1.15.1.1)
	General Reaction:
	2 Superoxide + 2 H^+^ = O_2_ + H_2_O_2_
Oxygenases	Catalyze the addition of oxygen into a substrate. They are further classified into:
	**Dioxygenase** (true oxygenases): These enzymes incorporate both atoms of molecular oxygen (O_2_) into the product(s) of the reaction
	Examples: Heme oxygenases (EC 1.14.99.3)
	**Mono-oxygenases** (pseudo-oxygenases; hydroxylases; mixed function oxygenases). These enzymes incorporate one oxygen atom as a hydroxyl group into the substrate, while the other oxygen atom is reduced to water
	Examples: cytochrome P450 enzymes (E.C. 1.14)
	General Reaction:
	Substrate_(reduced)_ + O_2_ = Product_(*oxidized*)_

Common redox-active centers include amino acid residues (e.g., tyrosine/cysteine), metal ions or complexes (e.g., Cu, Mo, Fe, Fe-S cluster, or Heme group), and coenzymes [e.g., flavin mononucleotide (FMN), flavin dinucleotide (FAD), pterin, and pyrroloquinoline quinone (PQQ)] (Younus, 2019). Oxidoreductase enzymes can act on a wide range of electron acceptors and electron-donating organic and inorganic substrates. In addition, the ones capable of generating radicals are potential biocatalysts for polymer synthesis (Xu and Kaplan, 2004), and cross-linking reactions between biopolymers.

Since so many biochemical reactions and industrial chemistry involve oxidation/reduction processes, the use of oxidoreductases to perform synthetic transformations has long been an attractive area of interest (May and Padgette, 1983). Their great biotechnological potential to participate in asymmetric synthesis of amino acids, steroids, other pharmaceuticals and specialty chemicals, as well as their clinical diagnosis and analytical applications have been recognized for many years. Back in the 80s, biotechnological applications in polymer synthesis and modification, pollution control and oxy-functionalization of hydrocarbons were already envisioned (May and Padgette, 1983). Therefore, the notion of developing practical biocatalytic applications of these enzymes has long been an important goal, although elusive. In fact, despite their great potential, oxidoreductases have been largely underused for biotechnological means. For large scale production of chemicals, the industry is not yet employing enzymatic oxidation reactions to a large extent. This is primarily due to the lack of enzymes with the desired selectivity, commercial availability and compatibility with the stringent process conditions (e.g., high concentrations of substrate, use of solvents, and strong oxidative conditions).

Furthermore, most commercially available enzymes are from mesophilic origin, obtained from microorganisms that grow optimally in moderate, narrow ranges of temperature and pH (i.e., between 20 and 45°C, neutral pH). However, given that industrial reaction settings often require high pressure, temperature and protein denaturing solvents, mesophilic enzymes usually underperform. For this reason, there is a considerable demand for more stable and better performing biocatalysts.

The main problems for the industrial implementation of oxidative enzymatic biocatalysts are currently being addressed via protein engineering, combining rational and computational design with directed evolution, to achieve the selectivity, catalytic efficiency and stability required for their industrial use (Martínez et al., 2017). In 2013, a 4-years collaborative Research and Technological Development Project called ‘‘Optimized oxidoreductases for medium and large scale industrial biotransformations’’ (INDOX; INDustrial OXidoreductases)^1^ was funded by the European Commission 7th Framework Programme (FP7). This research initiative aimed to coordinate efforts for engineering oxidoreductases of different families for applications in medium and large-scale oxidative biotransformations of industrial interest (Martínez et al., 2017).

Another solution that has proven to be efficient is taking advantage of what is already in nature by searching and discovering novel enzymes from extremophiles, as these microorganisms and their macromolecules have already adapted to thrive in environments that present extreme physicochemical conditions (e.g., high and low temperatures, acidic or alkaline pH, high pressure, salinity, among others) (Brininger et al., 2018). Consequently, extremophilic enzymes are more stable, active and robust than their mesophilic counterparts, allowing them to better perform biocatalysis under the harsh conditions found in most industrial applications (Caceres-Moreno et al., 2019). Working with extremophiles and their native enzymes, however, is not a trivial task due to the culture conditions required, their low cell yield and low enzyme expression. Therefore, in order to develop a novel extremozyme product, it is required to obtain a recombinant version of the enzyme of interest.

For the past 20 years, Fundacion Biociencia, a Chilean non-profit scientific research institution, has focused on the study of extremophiles and their bio-compounds, pioneering this area of Research and Development in Chile and Latin America. Five different types of extremophilic oxidoreductases that have been thoroughly studied at Fundacion Biociencia are discussed below and some of their interesting biotechnological applications are also explored.

## Laccases (EC 1.10.3.2)

Laccases are oxidoreductases that act on diphenols and related substances as donors, with oxygen as acceptor. These metalloenzymes belong to the protein family of multi copper oxidases (MCO) and are characterized by having four copper atoms that are essential for their catalytic function. These are divided into three types of structurally and functionally distinct copper sites, Type 1 (T1), Type 2 (T2), and binuclear Type 3 (T3), which can be distinguished by their unique spectroscopic features (Jones and Solomon, 2015). They catalyze the oxidation of a wide array of compounds; substrates for laccases include aromatic compounds (e.g., *ortho-* and *para-*substituted phenols, aromatic amines, N-heterocycles, aromatic thiols among others), metal ions and organometallics (Mate and Alcalde, 2017).

During catalysis, copper atoms mediate the electron transfer between the substrate and molecular oxygen, coupling the oxidation of the former with reduction of the latter (Jones and Solomon, 2015). The reaction results with water as the only secondary product, which is why laccases are considered “green” or sustainable biocatalysts (Riva, 2006).

The T1 copper atom is the closest one to the substrate binding site, directly accepting electrons from the reducing substrates. The redox potential of this copper atom is one of the main factors that influence the reactivity of laccases (Piontek et al., 2002). In accordance, laccases can only directly oxidize substrates with a lower redox potential than that of the T1 copper, but using redox mediators enables the indirect oxidation of other molecules (Claus, 2003). These are small diffusible electron carriers, which undergo rapid oxidation-reduction cycle at the active site of laccases leaving as free-radicals, which in turn oxidize molecules present in the reaction media that do not act as laccase substrates (Morozova et al., 2007). Free radicals generated during the reaction may also participate in other non-enzymatic reactions such as polymerization (Madhavi and Lele, 2009).

Some laccases isolated from thermophilic and hyperthermophilic microorganisms possess extreme thermostability, like the laccase from *Thermus thermophilus*, with a reported impressive half-life of 14 h at 85°C, reaching highest activity at 92°C and pH 5.5 (Miyazaki, 2005). The laccase from *Thermobacullum terrenum* possesses optimal temperature at 60°C with a half-life of 8 h at 80°C (Brander et al., 2015), while the laccase from *Thermus* sp. 2.9 retained 80% of its activity after 16 h at 70°C and after 6 h at 80°C (Navas et al., 2019). In addition, a novel laccase from the thermoalkaliphilic bacterium *Caldalkalibacillus thermarum* strain TA2.A1 showed optimum activity at alkaline pH 8.0 and 70°C, with a half-life of 12 h at 90°C, pH 8.0 and 6 h at 90°C, pH 9.0 (Ghatge et al., 2018). Halophilic laccases are also very attractive for biotechnological applications, two examples are a highly thermostable salt/solvent-tolerant laccase from the halophilic archaeon *Haloferax volcanii* (Uthandi et al., 2010) and the extracellular laccase produced by the halophilic bacterium *Aquisalibacillus elongatus* (Rezaie et al., 2017).

A novel and highly active spore-coat laccase was obtained from the thermoalkaliphilic bacterium *Bacillus* sp. strain FNT isolated from a hot spring in a geothermal site. The recombinant version of this enzyme shows optimal activity at 70°C and pH 6.0 with a half-life of 3 h when incubated at 60°C (Espina et al., 2021). This thermoactive enzyme has a very high specific activity and is currently commercially available as an enzyme product for the Research Market from Swissaustral LLC (Athens, GA, United States).

### Applications

Due to their broad substrate range, versatility and ease of use, laccases have great biotechnological potential and are useful in many applications as efficient and ecological oxidases.

Lignocellulosic biomass resulting from plant photosynthesis is the most abundantly available raw material on Earth, representing an enormous renewable source of energy, chemical products, and other materials. However, its usage is still limited due to its highly recalcitrant structure. Lignin as an insoluble complex polymer of phenolic compounds, is traditionally removed from wood by the process of delignification where chlorine- or oxygen-based chemical oxidants are conventionally used. However, they can cause serious problems in by-product disposal or cellulose fiber-strength loss. For this reason, enzyme-based delignification system is a much better alternative, and the use of laccases in conjunction with peroxidases [lignin peroxidase (LiP, EC 1.11.1.14), manganese peroxidase (MnP, EC 1.11.1.13) and versatile peroxidase (VP, 1.11.1.16)], cellulases and hemicellulases has an important synergistic effect on the degradation of the starting material, resulting in better yields of the desired products. Using laccases to produce bioethanol and paper pulp from plant biomass has been the subject of many studies, and the usage of thermophilic laccases has proven to be of particular value, as lignin and cellulose require high temperatures for its processing and degradation (Widsten and Kandelbauer, 2008).

Laccases have also been explored for the synthesis of polymers, as they can catalyze the crosslinking of lignin and phenolic compounds through the formation of free-radicals that react non-enzymatically to form ester or carbon-carbon bonds (Areskogh et al., 2010). This has been used for the polymerization of plant-polyphenolics. Tannins of higher molecular mass are known to have a longer circulation time *in vivo*, thus polymerization can be used to improve its biological benefits (Hagerman et al., 1998). Furthermore, laccase catalyzed co-polymerization and cross-linking allows for the functionalization of textiles, which has resulted in modified fabrics with antimicrobial, antioxidant or UV-protective properties (Sun et al., 2015; Sharma et al., 2019).

**FIGURE 1:**
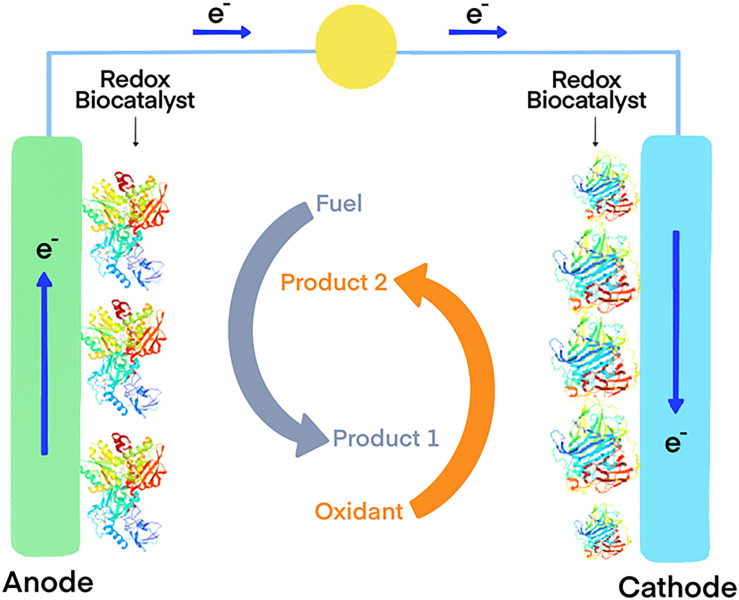
Model of an enzymatic biofuel cell that use oxidoreductase enzymes as electrocatalysts. The coupled oxidation of a fuel (typically reduced compounds such as glucose, and ideally organic waste produced by other industries such lignocellulosic biomass) and the reduction of an electron acceptor generates an electron flux that can be used to generate energy.

**FIGURE 2:**
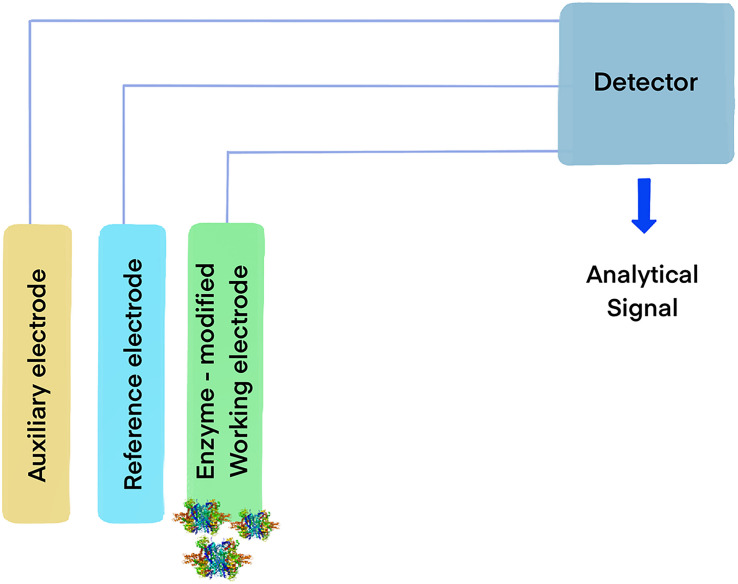
Model of an enzymatic biosensor based on oxidoreductases. The general mechanism consists in immobilizing an enzyme capable of oxidizing an analyte on the working electrode. When the enzyme catalyzes the reaction, the electrons yielded from the substrate generate an electrical signal in the detector, allowing for the identification of target molecules.

Another interesting possibility is the functionalization of carbon electrodes with laccases for biofuel cells (Figure 1) or biosensors (Figure 2) (Blanford et al., 2009). Real-world applicability of enzyme-modified electrodes is currently hindered by the intrinsic sensitivity to denaturation that most biocatalysts show, which negatively impacts the lifetime of the obtained constructs. Extremozymes, however, show notably higher stability compared to their mesophilic counterparts. This was used to advantage in an electrode prepared with the recombinant laccase from *Bacillus* sp. strain FNT produced at Fundación Biociencia. A laccase-carbon nanotube composite was produced, showing an evident increase in shelf-life compared to the equivalent conjugate produced using the laccase from *Trametes versicolor* (Atalah et al., 2018; Zhou et al., 2018).

In addition, laccases have immense applications in biodegradation of hazardous industrial soil/water pollutants, being currently an active area of research. Laccase catalyzed reactions can result in either direct degradation [e.g., dechlorination of chlorophenols, cleavage of aromatic rings, and mineralization of polycyclic aromatic hydrocarbons (PAHs; Madhavi and Lele, 2009)] or polymerization/immobilization (among pollutants themselves or copolymerization with other non-toxic substances). Then, easy removal of insoluble or immobilized pollutants can be achieved by adsorption, sedimentation, or filtration methods (Madhavi and Lele, 2009). Among persistent organic pollutants (POPs), synthetic dyes are of big concern since the resulting wastewater from dyeing processes has been rated as one of the most hazardous in terms of both volume and composition (Dave et al., 2015). To date, about 100,000 different dyestuffs are used industrially, with a worldwide production of nearly 800,000 tons per year (Schneider et al., 2004). Due to their complex structure, that ensures their durability and high color intensity, they are highly stable and very resistant to degradation (Li et al., 2019). The thermophilic laccase from *Bacillus* sp. strain FNT has been found to efficiently biodecolorize eight recalcitrant synthetic dyes from the three most important, structurally different types: azo, triarylmethane, and anthraquinone dyestuffs (Espina et al., 2021).

Furthermore, it has also been reported that laccases have the ability to effectively biodegrade chlorinated phenols from pulp paper mill effluents and black liquor as well as being able to detoxify a variety of xenobiotics such as PAHs (Librando and Pappalardo, 2013), endocrine disruptors (Barrios-Estrada et al., 2018), pesticides (Maqbool et al., 2016) antibiotics (Yang et al., 2017), biocides (Shi et al., 2016), fungicides (Yousefi-Ahmadipour et al., 2016), anticonvulsants (Ji et al., 2016b), anti-inflammatory drugs (Lloret et al., 2010; Singh et al., 2016), antidepressants (Tahmasbi et al., 2016), estrogen hormones (Lloret et al., 2010), lipid regulators (Ji et al., 2016a), insect repellents (Tran et al., 2013), sunscreen agents (Garcia et al., 2011), and even high energy materials (Ndibe et al., 2018).

## Hydrogenases (1.12.X.X)

Hydrogenases, also known as hydrogen dehydrogenases, are a large and diverse family of enzymes that catalyze the conversion of H_2_ into protons and electrons, and the reverse reaction producing dihydrogen. They contain complex metal centers at the active site, which are responsible for catalysis and are usually very sensitive to oxygen, causing many of them to undergo inactivation under aerobic conditions. Hydrogen can be seen as a universal energy source, supporting an immense variety of hydrogen-oxidizing microorganisms in a broad range of ecosystems. Accordingly, phylogenetic analyses have revealed that hydrogenases can be found in the genomes of microorganisms spanning the three domains of life and are present in most known environments. Hydrogen usage by microorganisms is generally part of an autotrophic or mixotrophic metabolism, granting survival in environments deprived of labile organic substrates. Physiologically, hydrogenases have the role of generating energy, providing reduced cofactors for the various reactions of the cellular metabolism, or dispersing reducing equivalents produced during fermentation (Constant and Hallenbeck, 2019). Based on the metal content of their active site, they can be classified into three broad classes: [NiFe]-hydrogenases, [FeFe]-hydrogenases, and Fe-hydrogenases (Lubitz et al., 2014).

[NiFe]-hydrogenases are composed of two structural subunits, a small, β-subunit (30 kDa) which can contain up to three iron-sulfur clusters that participate in electron transfer between the active site and a physiological electron mediator, and a large α-subunit (60 kDa) with four conserved cysteine residues coordinating the [NiFe]- active site. This type of hydrogenase is only found in archaea and bacteria, where they catalyze either hydrogen oxidation or production. Among them we can find the soluble hydrogenases produced by nitrogen-fixing cyanobacteria and bacteria (Bothe et al., 2010), the membrane bound hydrogen-producing enzyme first described in the hyperthermophile *Pyrococcus furiosus* (Sapra et al., 2003), and the tetrameric hydrogenases found in some hyperthermophiles such as *Thermococcus celer* (Blamey et al., 2001). Many hydrogenases from this family use NAD(P) or other soluble cofactors such as coenzyme F_420_. The sequences encoding these enzymes are commonly found on multiple operons containing the structural genes and some accessory ones, the latter of which are required for NiFe-hydrogenase maturation. This makes heterologous expression of [NiFe]-hydrogenases a very challenging task, with the incompatibility of the maturation apparatus of the host being a common pitfall. Nevertheless, heterologous expression of hydrogenase in closely related species, and in some cases, in *Escherichia coli*, has been reported (Constant and Hallenbeck, 2019).

[NiFe]-hydrogenases can be further classified in five different phylogenetic groups (Vignais and Billoud, 2007). Group 1 are membrane-bound hydrogenases that participate in hydrogen oxidation. These enzymes are key components of the electron transport chain of many microorganisms (Constant and Hallenbeck, 2019). [NiFe]-hydrogenases belonging to group 2 are cytoplasmic and include uptake hydrogenases found in nitrogen-fixing microorganisms that are crucial in protecting the nitrogenase against oxygen and hydrogen by recycling the hydrogen unavoidably generated as a by-product of nitrogen fixation (Bothe et al., 2010). The group 3 of [NiFe]-hydrogenases bind soluble cofactors such as NAD/NADP, and their activity *in vivo* is bidirectional. They are involved in electron transport energy and maintenance of redox balance in the cells. These hydrogenases include the well characterized bifunctional sulfhydrogenases of the anaerobic hyperthermophilic archaea *P. furiosus* (Ma et al., 2000). Hydrogenases of the group 4 are multimeric enzymes that reduce protons from water to dispose excess of reducing equivalents generated during fermentation. This group includes the hydrogen-producing enzyme first described in hyperthermophile *P. furiosus* (Schut et al., 2013). Group 5 NiFe-hydrogenase is comprised of the high-affinity hydrogen oxidating hydrogenase mainly detected in actinobacteria, Chloroflexi, and Acidobacteria (Constant and Hallenbeck, 2019).

[FeFe]-hydrogenases have been detected in bacteria and eukaryotes yet are not as widely spread in nature as NiFe-hydrogenases. The main function of most members of this diverse protein family is probably hydrogen production, and they interact with carbon metabolism to transfer electrons from reduced mediators (such as ferredoxin) to protons, yielding hydrogen (Constant and Hallenbeck, 2019). They can be found in many bacteria belonging to Clostridia, the best-known fermenters and hydrogen producers, and the organisms with the richest content of hydrogenases. In nature, the [FeFe]-hydrogenases are activated by a specific maturation machinery consisting of various auxiliary proteins, HydEFG, as well as the apoprotein, HydA (Nicolet and Fontecilla-Camps, 2012). Among hydrogenases, they possess an unsurpassed H_2_ production efficiency, owing to the structural properties of the active site cofactor, which comprises two distinct iron-sulfur clusters (Wittkamp et al., 2018). Some are monomeric, while other are multimeric, such as the ones from the hyperthermophiles *Thermotoga maritima* and *Caldicellulosiruptor saccharolyticus* (Van de Werken et al., 2008; Schut and Adams, 2009).

Fe-hydrogenases are homodimeric cytoplasmic enzymes, made of subunits weighting usually around 38 kDa. They are unevenly distributed across hydrogenotrophic methanogenic archaea and use hydrogen to provide reducing equivalents for the reduction of carbon dioxide into methane. They do not possess iron-sulfur clusters in their structure, do not mediate the reduction of one-electron acceptors [F_420_, NAD(P), or methyl viologen], and use hydrogen as a supplier of reducing equivalents instead of catalyzing the direct exchange between hydrogen and protons of water (Constant and Hallenbeck, 2019). They catalyze the reduction of H_4_MPT, a coenzyme that participates in methanogenesis.

### Applications

At this point there are enough climatic concerns about fossil fuel usage to make the need for newer and cleaner energy sources evident and urgent. Hydrogen has risen as a promising alternative, as it simultaneously has the highest gravimetric energy density of all known substances and undergoes combustion free of climate-active emissions or other undesirable residues (Møller et al., 2017). However, 95% of the hydrogen produced today is obtained from energy consuming methods that are still dependent on fossil fuels and hydrocarbon oxidation (Constant and Hallenbeck, 2019). The investigation of microbial hydrogen metabolism and its enzymes seems to be a promising alternative for biohydrogen production in a truly sustainable manner.

Biologically produced hydrogen can be obtained using hydrogenases. During the past decade, the complete conversion of glucose and xylose from plant biomass to H_2_ and CO_2_ has been made possible using an *in vitro* enzymatic pathway containing *P. furiosus* SHI [NiFe]-cytoplasmic hydrogenase alongside sugar reducing enzymes such as xylose isomerase, cellulase and glucose-6-phosphate dehydrogenase (Rollin et al., 2015). The difficulty of recombinantly expressing *P. furiosus* SHI, or obtaining it in sufficient quantities has been a hinder for real life applications of this enzyme, but in a recent work, through the overexpression of the maturation genes of SHI *Pyrococcus* hydrogenase, significantly higher yields were achieved, which makes the future production and application of the enzyme more feasible (Wu et al., 2018).

Taking advantage of hydrogen oxidation for energy production is also possible using biofuel cells (Figure 1). These devices use enzymes as electrocatalysts, usually immobilized in electrodes, to generate energy derived from a redox reaction. An example of this is the fuel cell constructed using a hyperthermophilic O_2_ tolerant hydrogenase and a thermostable bilirubin oxidase. The device delivered 15.8 mWh of power after 17 h of continuous operation (Mazurenko et al., 2017). In this application, hyperthermophilic enzymes greatly benefit from their intrinsic stability. A study comparing two bioelectrodes, one using the hydrogenase obtained from the hyperthermophile *Aquifex aeolicus* and the other the one from the mesophilic *Ralstonia eutropha*, demonstrated the superior stability of the hyperthermophilic one at high temperatures and at room temperature (Xiao et al., 2019).

Another interesting application for hydrogenases is cofactor regeneration. Most oxidoreductases require cofactors such as NAD(H) and NADP(H), which are expensive, and therefore retaining or recycling them *in situ* is of economic concern for the real-life application of these enzymes as catalysts, as it increases the total turnover number of the reaction. In light of this, the application of hydrogenases for NAD(P)H regeneration is an interesting perspective, as it uses dihydrogen as the substrate and only produces protons, which makes it compatible with other reactions taking place in a process. The hydrogenase *Ht*SH from the thermophilic bacteria *Hydrogenophilus thermoluteolus*, which was recently produced recombinantly in *R. eutropha*, is the first characterized [NiFe]-hydrogenase that showed to be able to perform H_2_-driven NAD^+^ reduction at high temperature and under aerobic conditions (Preissler et al., 2018). As such, it is an interesting candidate for *in situ* NADH regeneration.

Extremophilic microorganisms in general offer various advantages as a source for biotechnologically relevant hydrogenases. Halophilic hydrogenases, for example, are desirable because gas solubility is reduced as the salinity of the media increases. This lowers the levels of soluble O_2_, which as discussed above is a potent inhibitor of many hydrogenases, and also reduces the presence of dissolved hydrogen, which can exert product inhibition if it accumulates in the reaction media during biohydrogen production (D’Adamo et al., 2014).

In recent years, an efficient method for artificial [FeFe]-hydrogenase maturation has been developed, involving an artificially synthesized cofactor that readily inserts into the enzymatic binding pocket of the inactive apo-hydrogenase HydA1, generating fully functional [FeFe]-hydrogenases (Siebel et al., 2015). This discovery has allowed high-yielding production of active [FeFe]-hydrogenases, and site selective modifications of the cofactor. Eventually, the artificial maturation of [FeFe]-hydrogenases might be used to generate cofactor variants with novel catalytic properties and even novel enzyme reactivities. Some authors speculate that this could allow enantioselective hydrogenation reactions using modified hydrogenases as catalysts for the synthesis of pharmaceutical drugs or other compounds of interest, as well as enzyme variants of increased oxygen resistance and higher H_2_ production, allowing for the application of [FeFe]-hydrogenase in large scale industrial processes (Wittkamp et al., 2018).

## Glutamate Dehydrogenases (EC 1.4.1.2/1.4.1.3/1.4.1.4)

Glutamate dehydrogenases (GDHs) are oxidoreductases characterized by catalyzing the reversible oxidative deamination of glutamate into α-ketoglutarate and ammonia (Smith et al., 2019). They are classified depending on whether they accept NAD+ (EC 1.4.1.2) or NADP+ (EC 1.4.1.4) as a cofactor, with some enzymes accepting both molecules indistinctly (EC 1.4.1.3) (Ahn et al., 2000). Given its crucial role as a nodal point between carbon and nitrogen metabolism, GDHs can be found in almost all living organisms, with very few known exceptions (Hudson and Daniel, 1993; Plaitakis et al., 2017). Most known GDHs are homo-oligomers, although at least one hetero-oligomeric GDH is known (Tomita et al., 2010). Although bacterial GDHs were believed not to be allosterically regulated, the hetero-oligomeric GDH from the hyperthermophilic bacteria *T. thermophilus*, has shown to be allosterically activated by leucine and AMP, using a catalytically inactive adenine phosphoribosyltransferase as a sensory subunit for AMP activation (Tomita et al., 2019).

Known and characterized GDHs from extremophiles include the NAD^+^-dependent large homohexameric GDH from the psychrophilic bacteria *Janthinobacterium lividum* (Kawakami et al., 2007), the NAD^+^-dependent GDH from the extreme-halophile *Halobacterium salinarum* (Munawar and Engel, 2012a), which shows activity at temperatures up to 90°C and the stable GDH from the alkaliphile *Amphibacillus xylanus* (Jahns, 1996). Among thermophilic ones, the one produced by *P. furiosus* uses either NAD^+^ or NADP^+^ as cofactor and shows optimal temperature at 85°C with an impressive half-life of 10.5 h when incubated at 100°C (Diruggiero and Robb, 1995), while the recombinant version of GDH from *T. maritima* has optimal temperature at 75°C and a half-life of 1.8 h at 85°C (Knapp et al., 1997).

Archaea are a notable source of hyperthermophilic GDHs, such as the NADP^+^ dependent GDHs from *Thermococcus litoralis*, which has optimal temperature and pH at 95°C and pH 8.0 respectively, and 2 h half-life when incubated at 100°C (Ma et al., 1994). Other examples include the one from *Aeropyrum pernix* K1, with an optimal temperature of 95°C and optimal pH 7.0 with over 5 h half-life at 100°C (Helianti et al., 2001), and the GDH described in *Pyrobaculum islandicum* which shows higher activity at 90°C and pH 9.7 (Ohshima, 2012).

A novel thermostable NAD^+^ dependent GDH was purified from a thermophilic bacterium belonging to *Bacillus* genera isolated from Deception Island, Antarctica. The enzyme shows optimal activity at 65°C, pH 8.0 and a half-life over 8 h when incubated at 65°C outperforming the most important commercially available enzymes of its class (Flores et al., 2016). The recombinant version of this enzyme is currently commercially available as an enzyme product for the Research Market from Swissaustral LLC.

### Applications

Glutamate dehydrogenases have been extensively explored as biosensors (Figure 2) for medical diagnostic and the food industry, where the measurement of ammonia and glutamate in samples is important and can be determined monitoring the redox transitions of NAD(P)H in the presence of the enzyme and its substrates. The reaction between the compounds involved is equimolar, thus the analytes can be indirectly measured by observing the oxidation of NAD(P)H by the enzyme, in the presence of α-ketoglutarate using spectrophotometric or electrochemical methods (Atalah et al., 2019). This approach can be used to determine the levels of ammonia in clinical samples or in food, which is relevant in different contexts, such as hepatic damage diagnostic (Humbert et al., 2007), production of wine (Cheuk and Finne, 1984) and cheese (Calasso et al., 2020).

Immobilization of biomolecules on the surface of electrodes is a versatile method for the development of sensitive and selective electrochemical biosensors. These low-cost, easily portable, and fast responding devices enable the detection of small biomolecules including sugars, metabolites and amino acids (Matos-Peralta et al., 2019). The electrons produced by either the reductive amination of α-ketoglutaric acid or the oxidative deamination of L-glutamate catalyzed by GDH are transferred to the electrode, frequently involving a redox mediator such as hexacyanoferrate (III). The resulting change in current intensity causes an electrical signal correlated to the concentration of the analytes being measured. This technology has been used for the rapid and sensitive detection of α-ketoglutarate, using GDH immobilized on the surface of reduced graphene oxide-gold nanoparticle composite (Peng et al., 2020) and NH_4_^+^, using a voltammetric electrochemical biosensor constructed by immobilizing GDH onto a glassy carbon electrode on a Fe_3_O_4_/graphene/chitosan/nanocomposite (Yu et al., 2019).

Immobilized GDHs can also be used as enzymatic anodes for the construction of biofuel cells (Figure 1). Thermophilic enzymes are undoubtedly better suited for immobilization applications, since the mesophilic variants are reported to be too labile (Pasco et al., 1999). A recent example of this approach is the multi-enzyme bioanode built using the NAD-dependent GDH from the hyperthermophilic archaeon *P. islandicum*, along with three other thermophilic dehydrogenases (Satomura et al., 2019). This enabled a cascade reaction pathway for the efficient oxidation of L-proline. The bioelectrode yielded six-electrons for each molecule of substrate, and showed high durability at room temperature, indicative of great potential for applications in enzymatic fuel cells.

In the past decade GDHs have also served as a starting point for novel catalytic activities using protein engineering. Site directed mutagenesis allowed the conversion of the halophilic GDH from *H. salinarum* into a dehydrogenase accepting L-methionine, L-norleucine and L-norvaline as substrates (Munawar and Engel, 2012b). The enzyme was expressed heterologously in *H. volcanii*, and the resulting protein exhibited maximum activity at pH 10.0, enhanced thermostability and organic solvent tolerance at 70°C. Another example is a recent work in which a GDH from *Pseudomonas putida* was engineered by site-directed mutations to generate an enzyme capable of aminating 2-oxo-4-[(hydroxy)(methyl)phosphinoyl] butyric acid to produce the environmentally friendly herbicide L-phosphinothricin (Yin et al., 2018).

## Superoxide Dismutases (EC 1.15.1.1)

In the process of normal cellular metabolism, oxygen undergoes a series of univalent reductions, leading sequentially to the production of highly reactive oxygen-containing species (ROS) such as superoxide (${\text{O}}_{2}^{•-}$), hydroxyl (•OH) radicals and hydrogen peroxide (H_2_O_2_). They may cause oxidative stress by reacting with and damaging intracellular targets, such as lipids, proteins, and DNA. Consequently, all living species have developed systems to detoxify ROS, which include small antioxidant molecules and antioxidant enzymes (Fukai and Ushio-Fukai, 2011; Krumova and Cosa, 2016).

Superoxide dismutases (SODs), are ubiquitous to almost all forms of life and as their name suggests, they act on superoxide radicals as acceptors to catalyze its dismutation into molecular oxygen (O_2_) and hydrogen peroxide. This can be then further converted into harmless products by other enzymes like catalase and peroxidases (Miller, 2012).

They are metalloenzymes that are classified according to the metal centers they harbor in: Copper-zinc SOD (Cu(II)/Zn(II) SOD), manganese SOD (Mn(III)SOD), iron SOD (Fe(III)SOD), and nickel SOD (Ni(II)SOD) (Miller, 2012). Given that most SODs are strictly dependent on their cognate metal for catalysis, the binding of another one usually results in an inactive enzyme. However, due to the fact that Fe- and Mn-SODs coordinate their metal cofactor using the same protein ligands within an identical protein fold, there is a small group of the Mn/Fe-dependent SOD family that is active when loaded with either metal, and are known as cambialistic SODs, although the overall dismutation rate in cambialistic enzymes does not appear to be as optimized as that measured in the metal specific analogues, maintaining their metal selectivity (Abreu and Cabelli, 2010).

All SODs are very efficient biocatalysts that work by a similar mechanism in which the metal at the active site is reduced by one ${\text{O}}_{2}^{•-}$ and then reoxidized by the next ${\text{O}}_{2}^{•-}$. Therefore, the active site metal acts as a mediator passing an electron from one ${\text{O}}_{2}^{•-}$ to the next, so the electrostatic repulsion, which would prevent close approach of one ${\text{O}}_{2}^{•-}$ to another, is bypassed by the SODs (Fridovich, 2013).

Cu/Zn SODs are present in diverse locations in different organisms. They are found in the periplasm of bacteria, the cytoplasm and chloroplast of plants, and in several compartments such as the nucleus, lysosome, peroxisome, cytosol, and extracellularly in animals. This is because ${\text{O}}_{2}^{•-}$ is impermeable to the membrane, so it must be detoxified in the same compartment where it is formed (Bafana et al., 2011). Phylogenetically, they are clustered into two main subgroups of chloroplastic and cytosolic SODs (Fink and Scandalios, 2002). They are generally homodimeric, and as their name indicates, this class of enzymes contains both a copper and a zinc atom in each binding sites, which catalyze the reaction through the reduction of its two metal ions.

Mn SOD occurs in the cytosol of prokaryotes and the mitochondria, chloroplasts and peroxisomes of eukaryotes. Mitochondrial respiration releases significant amounts of ${\text{O}}_{2}^{•-}$ that need to be scavenged immediately, which makes this class of enzymes indispensable for eukaryotic life (Paul et al., 2007). This class of enzyme contains a manganese atom that cycles between Mn(III) to Mn(II) and back to Mn(III) as it goes about removing ${\text{O}}_{2}^{•-}$. The bacterial enzyme is usually a homodimer, whereas the mitochondrial enzyme is a homotetramer with some bacterial enzymes also being tetrameric. There is marked sequence similarity between bacterial and mitochondrial SODs, reflecting a close evolutionary history (Fridovich, 2013).

Fe SODs are found in the cytosol of bacteria, archaea and in the chloroplasts of plants. They are usually homodimeric or homotetrameric. In *E. coli*, its expression is constitutive, being found even in anaerobically grown cells, possibly being standby defense against ${\text{O}}_{2}^{•-}$, to protect in the event of a sudden exposure to O_2_. Fe and Mn SODs probably evolved from a common ancestor (Pilon et al., 2011).

Ni SOD was first isolated in 1996 from the cytosol of *Streptomyces* bacteria and since then, have been observed in other actinomycetes and cyanobacteria, as well as a few green algae species. They are homo hexameric proteins with each subunit’s active site containing a single nickel atom, and their mechanism involves a single electron transfer that is catalyzed by the Ni^2+^/Ni^3+^ redox couple (Shearer, 2014).

Superoxide dismutases have attracted wide research interest due to certain extraordinary biochemical properties. First, the electron transfer mechanism between the substrate and the enzyme active site is considered to have reached perfection, operating at close to the theoretical diffusion limit (Perry et al., 2009). Secondly, the enzyme from some sources shows unusual physicochemical properties such as stability to urea, freeze–thaw cycles, high temperatures, and prolonged refrigeration. For the last 40 years, approximately 30,000 research articles have been published on SOD and about 180 patents have been applied on the applications of SOD (Gopal and Elumalai, 2017).

It has been reported that SODs have been isolated from hyperthermophiles of the genera *Aquifex* (Lim et al., 1997), *Aeropyrum* (Yamano et al., 1999), *Sulfolobus* (Yamano and Maruyama, 1999), and *Pyrobaculum* (Whittaker and Whittaker, 2000). A thermostable Mn-containing SOD from *T. thermophilus* HB27, has been shown to be highly stable at 90°C and retained 57% activity after heat treatment at 100°C for 1 h (Liu et al., 2011). In addition, a thermostable Fe/Mn SOD was purified from *Bacillus licheniformis* strain SPB-13 isolated from thermal springs of the Himalayan region, the enzyme maintained 85% of its activity for 30 min and 75% of its activity after 2 h of incubation at 70°C (Thakur et al., 2019). Cold-adapted SODs have also been described, such as a Cu/Zn SOD from the psychrophilic bacterium *Halomonas* sp. ANT108 with optimum activity at 35°C, pH 8.0, and 13.9% activity at 0°C (Wang et al., 2020) and a Fe-SOD from the psychrophilic bacterium *Marinomonas* sp. NJ522, with optimal activity at 40°C, pH 8.0–10.0 and nearly 35% activity at 0°C (Zheng et al., 2006).

A novel thermostable SOD has been purified from a thermophilic bacterium, *Geobacillus wiegeli* (GWE1), isolated from a sterilization-drying oven at Fundacion Biociencia. The enzyme exhibited maximal activity at pH 8.5 and 60°C, with a half-life of 4.5 h after incubation at 60°C, and increased specific activity correlated with decreasing levels of superoxide when irradiated with UV-A, corroborating the direct involvement of this enzyme in the capture of ROS (Monsalves et al., 2013). The recombinant version of this enzyme is currently undergoing development.

### Applications

Due to its antioxidative effects, SOD has been widely applied in medical treatments as supplementation, where it helps to prevent or reduce the negative symptomatology of several conditions related to ROS-induced oxidative stress [e.g., atherosclerosis, aging, autoimmune diseases, cardiovascular diseases, cancer, diabetes, infertility, neurological disorders, transplant rejection, rheumatoid arthritis, asthma, and septic shock-induced tissue injury (McCord and Edeas, 2005)]. A Bovine- derived SOD commercialized under the name Orgotein, is used to treat the side-effects of inflammation and radiation. In addition, Chiron Corporation, acquired by Novartis, Switzerland, holds various patents for the recombinant production of human SODs, including various methods for the improvement of its thermostability, pharmacokinetics and catalytic efficiency (Gopal and Elumalai, 2017).

SODs are also widely used in cosmetics, commonly as a constituent of topical hair and skin care products where it aids wound healing, prevents hair graying, protects against UV rays, helps reducing facial-wrinkles, and promotes hair growth (Gopal and Elumalai, 2017). L’Oreal (France) were pioneers in the application of this enzyme for cosmetics and registered a patent for a marine source of SOD in 1973. To date, numerous prestigious brands such as Estee Lauder and Avêne have also developed formulations that include this enzyme as active ingredient (Bafana et al., 2011). The manufacture of cosmetics formulations often requires extended application of heat, thus thermostable SODs are desirable for this process.

Other applications include the utilization of SOD in biosensors for ${\text{O}}_{2}^{•-}$ detection (Tian et al., 2002), and for preservation of organs for transplantation (Nelson et al., 2005) and animal semen for livestock breeding (Zhang et al., 2017).

## Catalases (EC 1.11.1.6)

In cells, hydrogen peroxide is continuously produced enzymatically by the action of aerobic dehydrogenases, some oxidases and SODs, and non-enzymatically, as a side product of respiration or autooxidation of cell components (Krumova and Cosa, 2016). Excess H_2_O_2_ and the hydroxyl radical produced by its decomposition, are harmful for almost every cell component, being its rapid and efficient removal crucial.

Catalases are oxidoreductases that act on hydrogen peroxide as an acceptor (hydroperoxidases or peroxide reductases), characterized by utilizing a second H_2_O_2_ molecule as the electron donor, to catalyze its dismutation into water and oxygen without consuming cellular reducing equivalents (Heck et al., 2010).

There are some enzymes that exhibit not only catalase activity (EC 1.11.1.6) but also conventional peroxidase activity (EC 1.11.1.7), being able to also use other organic compounds, such as ethanol, as hydrogen donors. These bifunctional catalase-peroxidase enzymes have been classified under EC 1.11.1.21 as they have significant differences, including absence of sequence similarity and very different active-site, tertiary, and quaternary structures (Zamocky et al., 2008).

Typical catalases are ubiquitous enzymes found in Bacteria, Archaea, and Eukarya. They are homotetrameric enzymes, and according to phylogenetic analysis, there are three main clades that were segregated rather early in the evolution. Catalases from Clade 1 are found in bacteria, algae, and predominantly plants, and are characterized by having four small-subunits (55–69 kDa) and Heme B [protoporphyrin IX containing Fe(III)] prosthetic group. Clade 2 contains catalases from bacteria and fungi, with four large-subunits (75–84 kDa), Heme D and a “flavodoxin-like” domain. The most abundant subfamily found in prokaryotes, fungi, protists, plants, and animals is Clade 3, and includes catalases with four small-subunit (43–75 kDa) containing two distinct prosthetic groups: Heme B and NADPH as a second redox-active cofactor (Zamocky et al., 2008).

There is also a minor group of non-heme, manganese containing catalases (Mn-catalases) that were initially referred to as pseudo-catalases. These enzymes are present only in bacteria and archaea so far, and they have several differences with typical heme catalases. They have a homo-hexamer structure with four-helix bundle folds that confer them better stability at higher temperatures than heme-containing catalases. Also, they have a binuclear manganese complex in the active site (instead of mononuclear iron porphyrin). The H_2_O_2_ serves as an oxidant when a Mn(II)-Mn(II) state exists, and serves as a reductant when a Mn(III)-Mn(III) state exists (Chelikani et al., 2004). This makes Mn-catalases relatively sensitive to low H_2_O_2_ concentrations because its intermediate reaction is more stable and unlike heme catalases, they are not sensitive to cyanide inhibition of activity (Whittaker, 2012).

Currently, described catalases from extremophiles include the thermophilic Mn-catalases from *Geobacillus* sp. strain WCH70, which possesses optimal activity at 75°C and pH 9.0, with a half-life of 7.5 h when incubated at 80°C (Li et al., 2017), and from the archaeon *Pyrobaculum calidifontis* VA1, that has been found to possess optimal temperature at 90°C and half-life of 7.2 h at 90°C (Amo et al., 2002). A thermoalkali-stable heme catalase from *Thermus brockianus*, with optimum activity at 90°C and pH 8.0, and outstanding half-life of 14 days at 80°C (Thompson et al., 2003) and a typical catalase from the psychrophilic marine bacterium *Vibrio salmonicida*, with optimal temperature between 0 and 10°C and half-life of 6.5 h at 37°C (Lorentzen et al., 2006).

A novel catalase enzyme has been purified from a psychrotolerant Antarctic bacterium belonging to the *Serratia* genus that was resistant to UV-C radiation and well-adapted to cold temperatures. This microorganism, denominated strain I1P, was efficient at decreasing ROS levels produced after UV-C irradiation (Monsalves et al., 2020). The enzyme was found to be a heme catalase belonging to clade 3 and was active in a wide range of temperatures (20–70°C), showing optimal activity at 50°C and pH 7.0, with a half-life of 7 h at 50°C, which is remarkable considering its psychrotolerant origin. The recombinant version of this enzyme is currently commercially available as an enzyme product for the Research Market through Swissaustral LLC.

### Applications

Hydrogen peroxide is being extensively used as a powerful oxidizing, bleaching, or sterilizing agent in many industries, thus catalases have biotechnological potential and industrial importance.

The use of catalase in the food industry has already been approved by the U.S. Food and Drug Administration (FDA) and there are numerous patents available (Lončar and Fraaije, 2015). Hydrogen peroxide is widely used for cold pasteurization for the removal of microbiological contaminants present in beverages, and its removal has been achieved using catalase entrapped in alginate capsules (Trusek-Holownia and Noworyta, 2015). Among other applications, immobilized catalases have been used for the development of enzyme-based biosensors (Figure 2) that allow quick, sensitive and reliable monitoring of food quality and safety. They have been used for the detection of highly toxic chemicals in fruit juices, for detection of calcium in milk and water samples (Akyilmaz and Kozgus, 2009), for alcohol concentration in alcoholic drinks, and to determine bacterial contamination in food samples. In addition, catalases serve to scavenge free radicals responsible for food deterioration, as a food preservative, for the treatment of wrappers to prevent food oxidation and deterioration, and as antioxidant supplement believed to augment antioxidant defense systems of the body and help with the conditions that have been correlated to ROS-induced oxidative stress.

H_2_O_2_ is extensively used as a bleaching agent in the textile and paper industries. Traditional removal methods involve extensive washing (100 L of water/1 kg of textiles) that results in large volumes of alkaline wastewater, or the use of toxic chemicals that can cause further downstream problems. Enzymes are seen to have a vital role in reducing water consumption and pollution, as an example, the use of 1 Kg of Novozymes Terminox Ultra^>^ 50-L catalase preparation has suggested water savings of 20 m^3^/tonne of yarn, compared with the conventional method of rinsing to remove the hydrogen peroxide, reducing water consumption, energy waste, costs of chemicals plus saving time required for the process (Nielsen et al., 2009).

Even though these are very promising results that encourage the use of biocatalysts instead of traditional methods, they also reflect the need to develop more efficient enzymes, better suited to industrial processes. The amount of enzyme required to compensate for the loss of activity of a mesophilic enzyme could be largely reduced when using an extremophilic catalase, naturally more stable to the bleaching conditions.

Catalases have also been used for biomedical and clinical diagnostic applications. An enzyme complex [containing polyhemoglobin, SOD, catalase and carbonic anhydrase (PolyHb-SOD-CAT-CA)] has been developed as a blood-substitute also acting as an antioxidant and therapeutic agent against ischemia-reperfusion injuries (Bian et al., 2012). Therapy using catalase for inhibiting tumor growth has been implemented (Nishikawa et al., 2009) and a recombinant catalase has been used as immunomodulator for fighting H1N1 pneumonia (Shi et al., 2010). In addition, the utilization of catalase for contact lens cleaning solutions has been patented.

Finally, these enzymes can also be used for polymer synthesis, serving as biocatalyst to degrade residual H_2_O_2_ after a peroxide-triggered polymerization reaction (Lončar and Fraaije, 2015). This is very important as peroxide can compromise long-term storage of polyacrylate preparations or may interfere with polyacrylate formulations (Di Gennaro et al., 2014).

## Discovery, Development and Production of Novel Extremophilic Oxidoreductases

As already discussed, the versatility of applications, as well as their remarkable catalytic properties, make extremophilic oxidoreductases valuable and attractive biocatalysts with great biotechnological potential. The global demand and trade for industrial enzymes is steadily growing, and currently some suppliers distribute oxidoreductases for either industrial or research needs. Compared to hydrolases, significantly fewer oxidoreductases are commercially available today. Of those, only a small fraction is obtained from extremophiles. Table 2 presents some examples of the commercial laccases, hydrogenases, GDHs, catalases and SODs available, including few extremophilic enzyme products.

**TABLE 2:** Commercial oxidoreductases.

**Enzyme**	**Manufacturer**	**Brand name**	**Features**	**Uses**
Laccase	Swissaustral LLC	Laccase from Thermoalkaliphilic Bacterium	Obtained recombinantly in *E. coli*, functions at a wide temperature range, between 30–90°C, and pH 5.5–7.5	Research, pulp and paper industry, biodecontamination of water
	Sunsonzymes	Food grade laccase enzyme CAS 80498	Obtained from *Aspergillus oryzae.* Optimally active at temperatures around 50°C and pH 4.0–5.0	Food industry, bakery
		Laccase enzyme SQ10	Active in the range between 30°C and 70°C, pH 4.0–7.0	Removal of lignin from pulp or raw materials to improve pulp whiteness in the paper industry deinking, wastewater treatment
	Novozymes	Novozym^®^ 51003	Laccase from *A. oryzae*, functions optimally at 30°C and pH 7.5	Oxidizes various phenols, anilines, benzenethiols, metal ion complexes, and other compounds into quinones or other oxidized compounds
		Denilite^®^ IIS	Optimally active between 65–70°C	Textile
	ASA Spezialenzyme GmbH	Laccase A Laccase C	Active at temperatures between 50–60°C and pH 5.6 Performs best at 70°C and pH 5.0.	Precipitation of phenolic substances, enzymatic browning of food, gluing of flake boards, modification of elasticity and consistency of pastes, gums, production of microbiocides, analysis of phenols
Hydrogenase	Cytoplasmic [NiFe]-Hydrogenase SHI (Kerafast)	Cytoplasmic [NiFe]-Hydrogenase SHI (Kerafast)	NADP hydrogenase from the hyperthermophilic archaea *P. furiosus*, obtained recombinantly. Active from 25°C up to 100°C	Oriented for the research market
Glutamate Dehydrogenase	Swissaustral LLC	Glutamate Dehydrogenase from Thermophilic Bacterium	Obtained recombinantly in *E. coli*, Maintains over 85% of its activity for 8 h at 50°C, functions optimally at pH 8.0	Oriented for the research market, also applicable for diagnostic
	Roche	Glutamate Dehydrogenase [NAD(P)]	Functions in temperatures up to 60°C, with highest activity at pH 8.0	Diagnostic tests
	Sigma Aldrich	L-Glutamic Dehydrogenase (NADP)	Usable in the range between 45–65°C and pH 6.0–8.5	Diagnostic tests
Superoxide Dismutase	Creative Biolabs	rh-SOD	Human SOD produced recombinantly in *E. coli*	Oriented for the research market
	Creative Enzymes	Native *Bacillus stearothermophilus* SOD	Obtained from the thermophile *B. stearothermophilus*, shows no detectable decrease in activity up to 60°C, with optimal pH 9.5	Antioxidant with medical, cosmetic and nutritional applications
	Lonza	Biocell^TM^ SOD	Cu/Zn SOD obtained from yeast, temperature stability up to 45°C	Skin and hair care
	Jena Bioscience	Mn SOD^*His*^ Cu/Zn Superoxide Dismutase human, recombinant, *E. coli*	Human Mn SOD produced recombinantly in *E. coli.* Performs best at 37°C, pH 8.8	Oriented for the research market
Catalase	Genencor	Oxy-Gone^®^ T400	Active at temperatures ranging from 30 to 70°C, exhibits high level of hydrogen peroxide tolerance	Removal of hydrogen peroxide during textile processing before dying
	Swissaustral LLC	Catalase from Psychrotolerant Bacterium	Obtained recombinantly in *E. coli*, Maintains >75% activity in the range between 20 and 70°C	Research, clinical chemistry, textiles, waste treatment, cosmetics and as a disinfectant agent
	Sunsonzymes	Conzyme^®^ CAT50	Shows optimal activity at 50°C and pH 8.0	Textile processing and dyeing
	Novozymes	Terminox^®^	Functions at a wide range of pH and temperature up to 50°C	Removal of bleaching agents during textile dyeing

In spite of their great projection, there is still a way to go for real-life large-scale application of extremozymes as biocatalysts. Common challenges posed include the inherent difficulty of culturing and characterizing extremophilic microorganisms and their biomolecules, as well as the general lack of efficient systems of overexpression and purification. These challenges have been tackled from different angles by enzyme manufacturers and researchers.

On one hand, due to the difficulties and specific requirements of *in vitro* cultivation of extremophilic microorganisms, especially archaea, there is only a very small percentage of them that can be grown in a laboratory setting. Consequently, a culture independent approach based on metagenomic sequencing of environmental samples appears as a possible solution (Sysoev et al., 2021). However, the main disadvantages of this strategy are related to the correct identification of the target enzymes, since there is currently a lack of reliable functional annotation of extremophiles genomic data, caused by the low amount of experimentally described genes. This translates in a shortfall of specific databases, and therefore a great majority of the genes from extremophiles get annotated as hypothetical proteins with unknown functions, contributing to the problem of information shortage (Caceres-Moreno et al., 2019). In addition, even if an encoding gene of interest can be identified, it might not be fully functional once expressed, and it is not yet possible through genomic methodologies to precisely determine the specific biochemical properties of an enzyme. All these resulting in low detection and usage of extremozymes.

Further developments in sequencing, computation and bioinformatics might solve the problems associated with data mining for extremozymes and can help overcome the annotation difficulties in the near future. So far, we are currently seeing a boom of culture-independent methods for bioprospection of novel extremozymes from environmental metagenomes, which will help feed in the databases, opening the possibility to isolate and functionally characterize genes from unculturable extremophilic microorganisms (Khan and Sathya, 2018; Verma and Satyanarayana, 2020; Sysoev et al., 2021).

On the other hand, there is a culture dependent functional approach for the discovery and development of novel extremozymes, which has been largely used over the years with proven successful results (Caceres-Moreno et al., 2019; Espina et al., 2020). For this, culturable extremophilic microorganisms are isolated by means of traditional microbiology techniques from environmental samples taken during scientific expeditions to extreme environments. Then, enzymatic activity-based screening at desired conditions is performed to select promising candidates, and the native enzymes are purified from the isolated extremophilic microorganism and biochemically characterized. Subsequently, comprehensive bioinformatic analysis of the bacterial or archaeal sequenced genome allows to find the specific encoding gene of interest for its recombinant expression in a suitable heterologous host-vector system. After obtaining the recombinant extremophilic enzyme functionally overexpressed (either intra or extracellularly), it needs to be fully characterized to check the biochemical properties and compare them with the ones previously assessed in the native version (Caceres-Moreno et al., 2019).

In order to develop a novel enzyme product that could be produced and commercialized for the research market, it is necessary to perform growth optimization for quality-controlled scaling-up and downstream processing. Some of the parameters that need to be optimized in order to ensure satisfactory levels of biomass and protein overexpression are: culture media, pH, growth temperature, agitation rate, inoculum volume, optimum fermentation volume, culture time, antibiotic concentration (if needed) as well as inductor concentration, time, and temperature of induction (if required) (Espina et al., 2020). It is worth to note that achieving reproducibility in each batch in relation to biomass and/or protein production is very important, as the whole process must be validated to ensure that it will consistently generate a product meeting its predetermined specifications. Finally, the right package for an enzyme product must be also studied according to the format (lyophilized powder or liquid), stability in time at different temperatures, and storage conditions. If the final product complies with the expected Quality Control Certificate parameters and Product Datasheet specifications, it is labeled and delivered with all the necessary paperwork (e.g., Analysis Certificate, Material Safety Data Sheet and Certificate of Origin) to a final user or an appropriate distributor for its commercialization (Espina et al., 2020).

## Conclusion

Oxidoreductase enzymes mediate the redox reactions involved in biological systems for maintaining the structure and function of living cells. Consequently, they are one of the most abundant classes of enzymes within cells and their importance and great biotechnological potential has been recognized. Nevertheless, they have been largely underused, and enzymatic oxidation reactions are yet to be employed by the industry at large scale. This is primarily due to the lack of commercially available enzymes that match the requirements needed to operate under the harsh, strongly denaturing conditions of most industrial processes. This creates a demand for novel oxidoreductases and novel methods to produce them in sufficient quantities. Studying these enzymes to gain a complete understanding of their properties and mechanisms of action, as well as the organisms producing them, continues to be a relevant task.

Extremophilic oxidoreductase enzymes are naturally more stable, active, and resistant to denaturation than their mesophilic counterparts, as they are already adapted to function in extreme conditions. Moreover, the biochemical properties of extremozymes can be further improved by protein engineering, to better suit specific industrial needs. The novelty and degree of specification that can be achieved by further improving an already very competent enzyme is undoubtedly a better starting point for obtaining an ideal tailor-made biocatalyst product for a given industrial process.

The application of these enzymes in new manufacturing areas harnesses true potential to positively influence our relationship with the environment, opening the possibility of more sustainable industrial production models, where the replacement of chemical catalysts by enzymes will significantly reduce the environmental impact of industrial activity. Extremophilic microorganisms are a source of valuable biomolecules, and the study of their oxidoreductases can impact the future growth of industrial redox biocatalysis.

## Author Contributions

All authors listed have made a substantial, direct and intellectual contribution to the work, and approved it for publication.

## Conflict of Interest

The authors declare that the research was conducted in the absence of any commercial or financial relationships that could be construed as a potential conflict of interest.

## Publisher’s Note

All claims expressed in this article are solely those of the authors and do not necessarily represent those of their affiliated organizations, or those of the publisher, the editors and the reviewers. Any product that may be evaluated in this article, or claim that may be made by its manufacturer, is not guaranteed or endorsed by the publisher.

## Footnotes

1
http://indoxproject.eu
